# Comprehensive mapping of the effects of azacitidine on DNA methylation, repressive/permissive histone marks and gene expression in primary cells from patients with MDS and MDS-related disease

**DOI:** 10.18632/oncotarget.15807

**Published:** 2017-02-28

**Authors:** Magnus Tobiasson, Hani Abdulkadir, Andreas Lennartsson, Shintaro Katayama, Francesco Marabita, Ayla De Paepe, Mohsen Karimi, Kaarel Krjutskov, Elisabet Einarsdottir, Michael Grövdal, Monika Jansson, Asmaa Ben Azenkoud, Lina Corddedu, Sören Lehmann, Karl Ekwall, Juha Kere, Eva Hellström-Lindberg, Johanna Ungerstedt

**Affiliations:** ^1^ Center for Hematology and Regenerative Medicine, Department of Medicine Huddinge, Division of Hematology Karolinska Institutet, Karolinska University Hospital Huddinge, Huddinge, Sweden; ^2^ Department of Biosciences and Nutrition, Karolinska Institutet, Stockholm, Sweden; ^3^ Unit of Computational Medicine, Center for Molecular Medicine, Department of Medicine, Karolinska Institutet, Stockholm, Sweden; ^4^ National Bioinformatics Infrastructure Sweden, Stockholm, Sweden; ^5^ Molecular Neurology Research Program, University of Helsinki, and Folkhälsan Institute of Genetics, Helsinki, Finland; ^6^ Competence Centre on Health Technologies, Tartu, Estonia; ^7^ Department of Medical Sciences, Uppsala University, Uppsala, Sweden

**Keywords:** MDS, azacitidine, epigenetics, DNA methylation, histone modifications

## Abstract

Azacitidine (Aza) is first-line treatment for patients with high-risk myelodysplastic syndromes (MDS), although its precise mechanism of action is unknown. We performed the first study to globally evaluate the epigenetic effects of Aza on MDS bone marrow progenitor cells assessing gene expression (RNA seq), DNA methylation (Illumina 450k) and the histone modifications H3K18ac and H3K9me3 (ChIP seq). Aza induced a general increase in gene expression with 924 significantly upregulated genes but this increase showed no correlation with changes in DNA methylation or H3K18ac, and only a weak association with changes in H3K9me3. Interestingly, we observed activation of transcripts containing 15 endogenous retroviruses (ERVs) confirming previous cell line studies. DNA methylation decreased moderately in 99% of all genes, with a median β-value reduction of 0.018; the most pronounced effects seen in heterochromatin. Aza-induced hypomethylation correlated significantly with change in H3K9me3. The pattern of H3K18ac and H3K9me3 displayed large differences between patients and healthy controls without any consistent pattern induced by Aza. We conclude that the marked induction of gene expression only partly could be explained by epigenetic changes, and propose that activation of ERVs may contribute to the clinical effects of Aza in MDS.

## INTRODUCTION

Myelodysplastic syndromes (MDS) constitute a group of clonal, heterogeneous bone marrow diseases characterized by disturbed myeloid differentiation and a propensity for clonal evolution and leukemic transformation. Recurrent mutations in genes encoding e.g. epigenetic regulation, splicing and signaling are essential in the pathogenesis of MDS and may contribute to the aberrant expression profiles described in the disease [1–4]. However, it has since long been widely assumed that the aberrant expression profiles and defect differentiation in MDS and in leukemic progression are closely related to the marked DNA promoter methylation reported in several studies [4–7].

Azacitidine (Aza) is a cytosine analog and an inhibitor of DNA methyl transferase (DNMT) licensed in Europe as first-line treatment for higher-risk myelodysplastic syndromes, in the US for all types of MDS and frequently used also in other myeloid malignancies, such as acute myeloid [8, 9] Treatment has been confidently associated with prolonged survival in these patient groups [10]. Aza treatment results in reduced DNA methylation as demonstrated by several studies *in vivo* and *in vitro*, although the degree of demethylation seems to be limited [4, 11–15]. A plethora of *in vitro* studies have investigated the *in vitro* effects of Aza, however, only a limited number of these have used primary MDS cells in the experiments [16]. In a previous study we observed a demethylating effect of Aza on primary MDS progenitors, and a reduction in H3K9ac and H3K27ac, reflecting a more complex effect of Aza on the epigenome than previously believed [4]. Other studies have investigated methylation status in patient bone marrow before and after Aza treatment. Some of these demonstrated a prognostic beneficial effect of an initial reduction in DNA methylation, but whether this reflects a causative effect or only an association is unclear [12, 13, 17–19]. A couple of studies have shown an association between pre-treatment DNA methylation profiles and response, while others find no such association [13, 20–22]. It is important to note that these studies most commonly evaluate promoter methylation, and do not take into account changes in methylation outside the promoters. Thus, in order to predict which patients will respond to Aza it is vital to understand its mechanism of action on primary MDS cells.

The link between DNA methylation and transcription as a basic biological principle is well established, although promoter DNA methylation does not always correlate to gene expression. In fact, in AML, there is only a moderate correlation between DNA methylation and transcription [23–25]. There is hitherto no clear evidence that changes in gene expression observed during Aza treatment results from changes in DNA methylation. A few studies have investigated the genome-wide effects of Aza treatment *in vitro* on DNA methylation and gene expression in various cell lines. In HEK 293 cells, only a small minority of genes upregulated by Aza could be explained by changes in DNA methylation, and vice versa in hypermethylated colon cancer cells; only 1,6% of genes demethylated by Aza gained chromatin accessibility [26, 27]. Additional studies confirm a decrease in DNA methylation by Aza but show modest effects on gene expression and a lack of association between demethylation and gene expression [28–31].

Two papers from 2015 showed that in solid tumor cell lines, Aza induces expression of endogenous retroviruses (ERVs), formation of dsDNA and a subsequent activation of the innate immune system after long time culture [32, 33].

This is the first study to simultaneously assess the global genome-wide effects of Aza on DNA methylation, the histone modifications H3K9me3/H3K18ac, and gene expression in MDS bone marrow progenitors *in vitro*. Aza-treatment of MDS CD34+ progenitors resulted in a genome-wide but modest DNA demethylation predominantly in heterochromatin, and was accompanied a similar change in H3Kme3. In line with what has previously been demonstrated in cell lines, we observe an activation of ERVs in these primary MDS progenitors. Furthermore, we showed that Aza induces a general increase in gene expression, although only to a minor extent explained by epigenetic changes, however partially explained by an increase in transcription factors affecting differentiation.

## RESULTS

### Patient population

Bone marrow samples were collected from 11 patients with a clinical indication for Aza treatment; (6 higher-risk MDS, 4 AML with multilinear dysplasia and 20–29% blasts and 1 CMML-2). Patient characteristics are shown in Table 1. One patient sample did not yield enough cells for further experiments (data not shown), and ten patient samples thus proceeded to the culture experiments. All samples were used fresh without a previous freezing/thawing procedure. Analyses on DNA methylation, gene expression H3K9me3 and H3K18ac was successful in 9, 4, 6 and 2 patients, respectively.

**Table 1:** Patient characteristics

Patient identity	WHO	Marrowblastcount	Cellularity	Hb	WBC	ANC	Plt	Transfusiondependency	Cytogenetics	IPSS	Mutations	Treatment	Response
1	RAEB-II	11	70	89	1.1	0,2	99	Yes	Normal	Int-2	TP53	Intensive chemotherapy	Complete remission
2	RAEB-I	6	70	104	2.9	1.4	69	Yes	Complex	High	DNMT3A; SF3B1;TET2	Supportive care	Never evaluated
3	RCMD	3	90	91	3.7	2	92	Yes	Complex	Int-2	No mutation	Azacitidine + Lenalidomide	Complete remission
4	RAEB-II	10	50	95	2.2	0.8	103	No	Del5q	Int-2	No mutation	Azacitidine	Never evaluated
5	AML w multilinear dysplasia	28	80	110	2.3	0.9	43	No	Complex	High	IDH1;TET2;	Azacitidine	Complete remission
6	AML w multilinear dysplasia	17	80	83	10.4	2.9	255	Yes	Normal	High	RUNX1;	Azacitidine	Progression
7	AML w multilinear dysplasia	23	80	130	1.8	0.5	85	No	Normal	High	No mutation	Azacitidine	Complete remission
8	CMML	14	100	104	49.1	31.8	11	Yes	Normal	NA	ASXL1;KRAS;PRPF40B;RUNX1;U2AF1;	Azacitidine	marrow Complete remission
9	RAEB-I	9	30	111	1.6	0.4	196	Yes	Complex	Int-2	TP53;TP53;	Azacitidine	Stable disease
10	RAEB-II	14	80	93	2.4	0.4	63	Yes	-Y	Int-2	SRSF2; TET2;TET2	Azacitidine	Hematological improvment
11	AML w multilinear dysplasia	28	60	100	4,1	1,2	86	No	Complex	High	CREBBP; MLL; NRAS	Intensive chemotherapy	Stable disease

Abbreviations: RAEB-II Refractory anemia with excess of blasts. RCMD refractory cytopenia with multilinear dysplasia, AML acute myeloid leukemia, CMML chronic myelomonocytic leukemia, WBC white blood cell count, ANC absolute neutrophil count, IPSS international prognostic score system

### Cell culture growth and viability

Aza moderately decreased cell growth in the cultures compared to cells cultured without Aza; median cell growth for Aza treated samples at 24 h was 140% (range 65-333%) compared to control samples (196%, range 87-390%), thus there was no significant change in cell growth upon Aza treatment (Supplementary Figure 1). At continued culture up to 48 hours, without additional Aza, both control and Aza treated cells discontinued growing and started dying, and cell count was 129% (range 100-283%) of baseline for control and 98% (range 86-318%) of control for Aza treated cells.

### Correlation between gene expression and epigenetic marks in untreated cells

We observed an inverse correlation between DNA methylation and gene expression (rho −0.305, p=9.6×10^−197^; Spearman's rank test), showing a significant correlation between gene-specific DNA methylation and gene expression in MDS CD34+ cells. Additionally, DNA methylation correlated significantly to the active chromatin mark H3K18ac with an inverse correlation (rho −0.318, p<10^−200^), and to the repressive chromatin mark H3K9me3 (rho 0.144, p=4.5×E^−80^) indicating that DNA methylation and chromatin marks interplay to regulate gene expression, and that genes with closed chromatin also are highly DNA methylated. Active H3K18ac and repressive H3K9me3 chromatin marks also correlate inversely to each other (rho −0.152, p=3×10^−174^). In addition, there was a correlation, although less pronounced, between chromatin marks and gene expression (rho −0.06, p=6×10^−9^ for H3K9me3; and rho 0.083, p=3.5×10^−16^ for H3K18ac).

### Increased gene expression after Aza exposure

**Figure 1:**
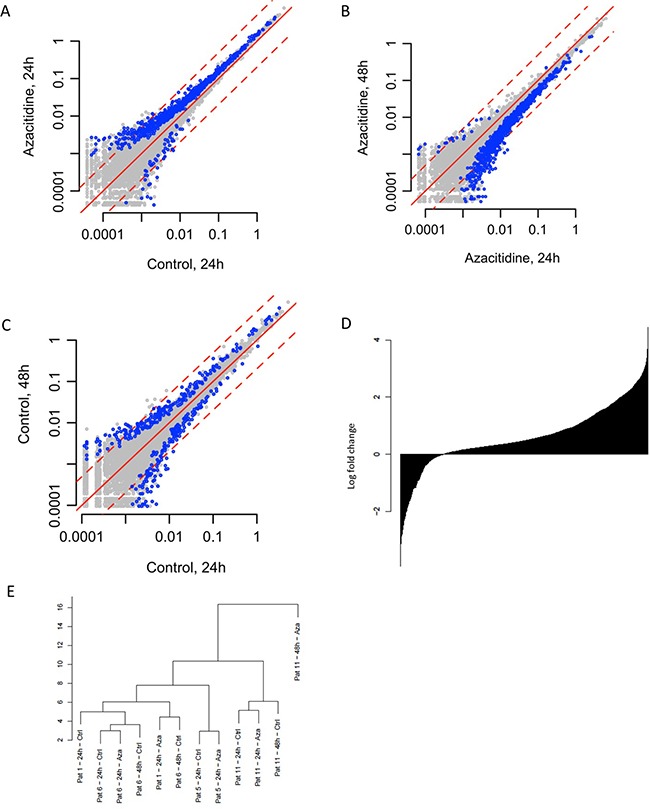
Effects of Aza on gene expression **(A)** Differences between Aza 24h culture vs. control 24h culture **(B)** Aza 48h vs. Aza 24h **(C)** Control 48h vs. control 24h. **(D)** Change in gene expression; all genes sorted from most negatively to most positively changed and printed as a barplot. **(E)** Unsupervised clustering of gene expression data.

Comparison of cells cultured without Aza for 24 h vs. 48 h (paired samples from 2 patients), showed no global changes in gene expression, with 5011 and 4135 genes being up and down regulated, respectively, indicating that the culture conditions per se did not influence the results (Figure 1A).

Comparing treated and untreated cells cultured for 24 h (paired samples from four patients), we observed a general increase in global gene expression among the Aza samples, with 7444 and 2930 genes up and down regulated, respectively; whereof 924 and 54 with p-value <0.05, (Figure 1A and 1D). The median fold change was 1.61 (q1=1.16; q3=3.32). Global gene expression increased in all four patients (data not shown).

Cells cultured with Aza for 24h, followed by another 24 hours in culture without Aza showed a general decrease in gene expression between 24 and 48h, with 2038 and 7336 genes up and downregulated, respectively (Figure 1B). Out of the genes 7444 genes upregulated in the 24h Aza samples, 5952 (80%) genes had decreased expression in the 48h Aza samples, indicating a rebound effect. Similarly, out of the 2930 genes downregulated in the 24h Aza samples, 949 (32%) showed increased expression in the 48h Aza samples. GO analysis of upregulated genes at 24 h Aza culture showed enrichment of genes involved in translation, RNA processing and ribosomal function (Supplementary Table 2).

Samples clustered together mainly based on patient identity, and not based on whether treated with Aza or not (Figure 1E).

### Aza induces a general but limited DNA demethylation

DNA obtained from CD34+ bone marrow cells allowed for assessment of differences in global methylation between samples cultured with or without Aza for 24 h (n=9), freshly isolated samples and cells cultured without treatment for 24 h (n=4), samples cultured without treatment for 48 h (n=2) and samples cultured with Aza for 24 h or 48 h (n=4). The density plot in Supplementary Figure 2 reveals an even distribution of β-values across the samples.

**Figure 2:**
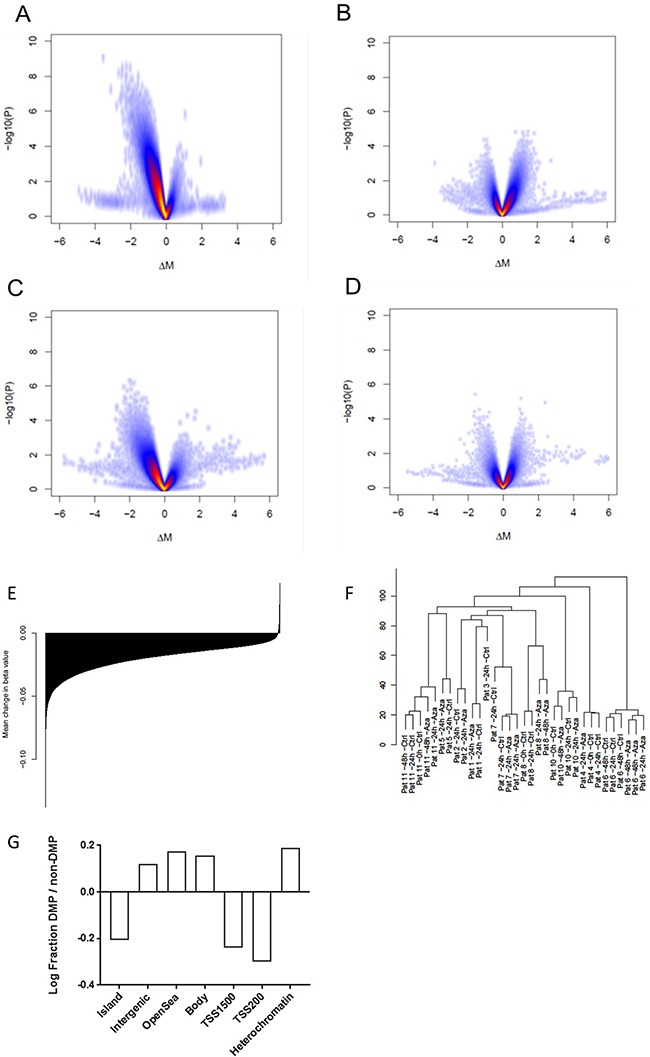
Effects of Aza on DNA methylation **(A)** Volcano plot illustrating differences between Aza 24h vs control 24h. **(B)** Aza 48h vs 24h **(C)** Aza 48h vs control 48h **(D)** Control 24h vs no culture **(E)** Mean change in methylation for all probes annotated for specific genes; all genes sorted from most negatively to most positively changed and printed as a barplot. **(F)** Unsupervised clustering of methylation data **(G)** Log fraction of differentially methylated probes (DMPs) divided by non-DMPs in different areas of the genome ass annotated by the Illumina 450k array (relation to CpG island and promoter) and epigenomic roadmap (heterochromatin).

No effect of the cell culture condition on global DNA methylation pattern was identified in the untreated cultures at 0 h and 24 h, indicating that culture conditions per se did not alter methylation status (Figure 2D), despite the fact that cells were proliferating, see Supplementary Figure 1.

We identified 65 769 differentially methylated probes (DMPs) comparing samples cultured with or without Aza for 24h; the vast majority of these (n=65 664) were less methylated in the Aza treated samples (Figure 2A). The relative proportion of DMPs was lower in CpG islands compared to the Illumina 450k reference while the proportion was higher for open sea probes (Figure 2G). Similarly, the proportion of DMPs compared to the reference was smaller for probes located in proximity to transcription start sites while it was higher for probes located in gene bodies or non-gene related probes, (Figure 2G). Moreover, comparing the DMP proportion in relation to the epigenome roadmap of primary CD34+ cells, we observed an enrichment of DMPs in heterochromatin defined by H3K9 methylation in our material (Figure 2G) [34]. The relative median change in β-value per probe, comparing Aza- and control samples, was −0.018 (q1=−0.035; q3=−0.007) while the relative change per gene, calculated as mean change for all probes annotated to specific genes, was −0.018 (q1=−0.026; q3=−0.012), thus a very modest reduction (Figure 2E). In total, 20124 and 148 genes showed decreased and increased DNA methylation, respectively. When looking only at promoter-associated probes (TSS-1500bp), the median relative change per gene was β −0.015. All patients except one showed a global decrease in DNA methylation; see patient-specific effects in Supplementary Figure 3. We then calculated the degree of DNA demethylation on our previously published dataset which was generated using the same methodology [4]. Indeed, the degree of DNA demethylation was very modest and at the same levels as in the present study with a mean decrease in β -value of 0.024 and 0.012 in MDS CD34+ cells and healthy control CD34+ cells, respectively.

The 48 h Aza samples showed a general increase in methylation compared to the 24h Aza samples, although no significant DMPs were identified (Figure 2B). Whether this reflects changes at the single cell level or changes in cell composition due to proliferation remains to be investigated. The total effect of Aza over 48h, comparing 48h Aza samples with 48h untreated samples was a global reduction in DNA methylation (Figure 2C).

An unsupervised clustering analysis showed strong clustering based on patient identity rather than on treatment or culture conditions (Figure 2F). We could not find any clustering according to mutation pattern, cytogenetics, blast count, diagnosis or clinical treatment response (data not shown).

To assess whether DNA demethylation was more profound in the CD34- compared to the CD34+ progenitor fraction, vital frozen MNC from five MDS patients were cultured for 24h with or without Aza, then separated into CD34+ and – fractions and assessed for DNA methylation with the Illumina 450k array. The CD34- cells were however even less demethylated than CD34+ cells from the same experiment (change in β value of −0.008 vs −0.027), which contradicts the potential explanation that Aza improves the hematological condition mainly by acting on more mature precursor cells, see Supplementary Figure 4, 5 and 6.

### Changes in active chromatin mark H3K18ac

**Figure 3:**
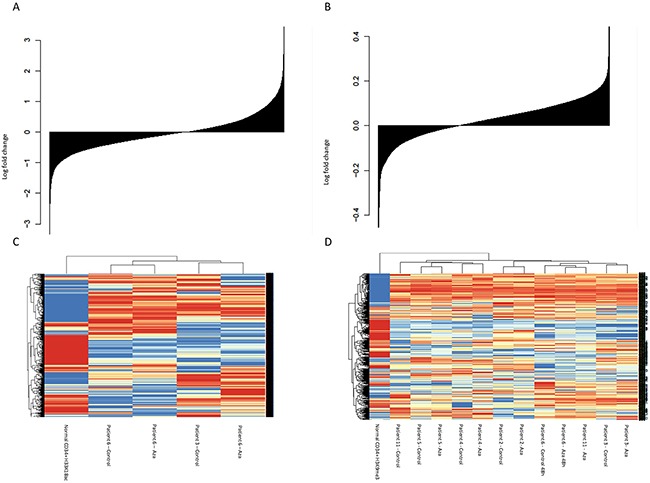
Effects of Aza on histone modifications **(A)** Change in H3K18ac; all genes sorted from most negatively to most positively changed and printed as a barplot **(B)** Change in H3K9me3c **(C)** Heat map and clustering of patients based on H3K18ac **(D)** Heat map and clustering of patients based on H3K9me3.

Analysis of H3K18ac, comparing cells cultured for 24h with or without Aza, was successful with more than 10 million reads in 2 patients. The median fold change between Aza and control samples for peaks of H3K18ac after peak calling was 1.01 (q1=0.95; q3=1.07), as shown in Figure 3A. There was avariation in H3K18ac pattern between the two untreated samples, differences that remained in the Aza samples, and normal CD34+ cells clustered separate from MDS CD34+ cells with regard to H3K18ac pattern (Figure 3C). A significant difference between normal CD34+ and MDS CD34+ cells was found in 1203 genes, with mean number of reads = 94. No GO pathways were significantly enriched.

### Changes in repressive mark H3K9me3

Analysis of H3K9me3 patterns, comparing cells cultured for 24h with or without Aza, was successful with more than 10 million reads per sample in 6 patients. Median fold change between Aza and control samples for peaks of H3K9me3 after peak calling was 1.09 (q1=0.71; q3=1.67), see Figure 3B. Similar to the patterns for H3K18ac, healthy CD34+ cells clustered separately from MDS patients samples with regard to H3K9me3 pattern (Figure 3D).

Included genes in the heat map were 504 genes with a significant difference between normal CD34+ and MDS CD34+, with a mean number of reads of 28. Unsupervised clustering of H3K9me3 data showed clustering mainly based on patient identity (Figure 3D).

No GO pathways were significantly enriched.

### Correlation between change in epigenetic marks and gene expression

There was a highly significant correlation between changes in the repressive chromatin mark H3K9me3 and changes in DNA methylation (rho 0.136, p= 1.7×10^−69^). Interestingly, by analyzing patientwise correlation (n=6), we observed a strong correlation reflected in very low p-values for each patient, see Supplementary Table 3. Interestingly three patients each showed a positive and a negative correlation, respectively, without any association disease characteristics e.g. cytogenetics, morphology or mutational status (data not shown). Moreover, we observe a weak correlation between H3K9me3 and gene expression (rho 0.053, p=2.38×10^−7^) which only can explain a minor part of the gene expression variation. No other epigenetic marks correlated with gene expression or with another epigenetic mark (H3K9me3 and H3K18ac).

### Motif analysis

Analysis of common transcription factor binding motifs in the 924 significantly upregulated genes in the Aza samples compared to non-treated samples showed enrichment of binding motifs for eight transcription factors that were themselves upregulated more than 1.8-fold (median), namely *ELK1, STAT 1, STAT3, RUNX1, GABPA, ERG, NRF1* and *PU.1*. Several of these transcription factors regulate differentiation, indicating that Aza indeed may affect hematopoietic stem/progenitor cell differentiation. Interestingly, the active chromatin mark H3K18ac was already high in these genes and did not increase in the Aza treated samples. Neither did DNA methylation change significantly in any of the genes encoding for those transcription factors (median reduction of β-value of 0.011). Hence, changes in epigenetic regulation, as measured by H3K18ac and DNA methylation, cannot explain the increased expression of the TF, and the upstream regulation remains unknown.

### Aza induces expression of transcripts containing ERV-like repetitive elements

By overlapping gene expression data with regions annotated as repetitive elements, we observed 16 TFEs overlapping with repetitive elements, being significantly (p<0.05) upregulated in the Aza treated samples, while only three were significantly downregulated. Of the upregulated repetitive elements, 15 belong to the ERV family (Supplementary Table 4 and Supplementary Figure 7). No Illumina 450k probes were annotated to the ERV-associated regions and the change in DNA methylation in these regions could therefore not be analyzed. We observe no general reduction of H3K9me3 peaks in the Aza samples compared to control samples over the ERV regions (data not shown).

## DISCUSSION

To better understand the effect of Aza on epigenetic marks and transcription profiles in MDS progenitors, we set out to assess changes in DNA methylation, chromatin marks and gene expression in primary MDS patient CD34+ cells, after short term incubation with Aza. The initial hypothesis was that promoter DNA demethylation would lead to gene expression, but that an increase in repressive chromatin marks may counteract this process [4]. Indeed, we found a significant increase in global transcription levels in all patients after 24 h Aza exposure. Upregulated genes were mainly involved in GO pathways including translation, RNA processing and ribosomal function. As expected, we detected a general, significant DNA demethylation following Aza treatment, however, the degree of demethylation in each specific gene was very modest, with a mean reduction of β-value of only 0.018, and thus of questionable biological relevance. The DNA demethylation occurred in all genomic regions, but to a larger extent in non-promoter regions, open-sea and in regions annotated as heterochromatin, supporting the interpretation that this was not the direct cause of increased gene expression. This was also supported by the lack of correlation between changes in expression and DNA methylation at the individual gene level.

We next searched for other epigenetic explanations for changes in gene expression and assessed if Aza treatment affected active (H3K18ac) or repressive (H3K9me3) chromatin marks, and found that changes induced by Aza in H3K9me3, which is strongly associated with heterochromatin formation, exhibited a weak correlation to the Aza induced global changes in gene expression while H3K18ac showed no such correlation with change in gene expression. Moreover, we observed a strong correlation between change DNA demethylation and change in H3K9me3, indicating that Aza may have a profound effect in heterochromatin regions, defined by H3K9me3 enrichment. The reason for the paradoxical observation of three patients with positive, and three with negative correlation between DNA demethylation and change in H3K9me3 is unclear. Hypothetically, differences in epigenetic baseline pattern and mutational status of histone modificating enzymes might explain the differences. In line with these observations, a previous publication reports different Aza-induced effects on histone modificaitons depending on what type of cell line is used, indicating that different cells might respond epigenetically different to Aza [35]. There was a tight correlation between epigenetic marks defining heterochromatin e.g. DNA methylation and H3K9me3 in the untreated samples. Additionally, there was a significant baseline correlation between all epigenetic modalities and gene expression, indicating high quality and robustness of our data.

As the marked induction of gene expression only could partly be explained by epigenetic changes, we analyzed TF binding motifs of the significantly upregulated genes, and found eight transcription factors significantly enriched for binding sites in these genes, all of which were themselves upregulated in expression. Thus, at least part of the upregulated genes may be explained by Aza-induced increase of the above TFs. Since several of them have important regulatory functions in hematopoietic differentiation, interference with the differentiation process might be part of how Aza exerts its effect. Our epigenetic data cannot, however, explain the change in expression of these TF and the upstream events remain to be further investigated.

Interestingly, we observed an activation of 15 ERVs in the Aza treated samples. Recent publications in cell lines have demonstrated an early Aza-induced activation of ERVs, followed by a later activation of immune response genes [32, 36]. We show for the first time that Aza treatment results in ERV activation also in primary CD34+ MDS cells. The short incubation time in our experiment did not, however, allow us to study delayed upregulation of immune response genes, as long-term incubation of primary CD34+ MDS cells is not possible due to cell differentiation and reduced viability beyond 48 hours of culture [4]. Although we have not analyzed DNA methylation over these repetitive elements, activation is consistent with the documented role for DNA methylation in silencing of retrotransposons in mouse embryonic stem cells [37]. It is well established that global DNA demethylation during mammalian gametogenesis and early embryonic development results in the de-repression of ERVs and this correlates with the pluripotent capacity [38, 39], and differential ERV expression is involved in embryonal cell differentiation [40] Thus it is possible that the increased expression of ERVs we observe in MDS cells could contribute to changes in cell differentiation upon treatment with Aza.

In contrast to previous studies on cell lines and primary AML cells, we detected a general increase in polyadenylated RNA induced by Aza, as assessed by RNA sequencing. One possible explanation for this novel observation compared to other studies on non-MDS cells is that MDS progenitors might be more susceptible to Aza. Another is that the spike-in normalization RNA sequencing method used in our study might yield higher sensitivity compared to the array-based gene expression methods used in other studies. Primarily, we propose that the increased mRNA levels are due to increased transcription. However, although previous studies indicate that Aza leads to reduced stability of RNA and increased degradation [41–43], we cannot exclude the possibility of decreased degradation of mRNA.

The current study is the first to comprehensively investigate the associations between epigenetic modalities and gene expression in Aza-treated primary MDS bone marrow cells. We demonstrate a complex effect of Aza on the epigenome and the transcriptome which gives important information in the search for how Aza exerts its effect. However, the link between the epigenetic modalities and gene expression is only partly explained in our study and further studies mapping other histone marks, and also mapping DNA methylation using methods with higher resolution e.g. bisulphite sequencing are warranted. The role of the TFs and ERVs identified as activated in our study needs to be studied further in functional assays as well as in an *in vivo* setting.

## METHODS

### Patient population

Bone marrow samples from consecutive patients with an indication for Aza treatment, i.e. MDS with International Prognostic Score System (IPSS) intermediate-2 or high, Chronic myelomonocytic leukemia (CMML) with >10% blasts or Acute myeloid Leukemia (AML) with multilinear dysplasia and <30% blasts, were collected [44]. All patients were treated in clinical routine practice at the Karolinska University Hospital. Samples proceeded to cell culture without preceding freezing/thawing. The study was approved by the Ethical committee (Dnr 2010/427-31/1) at Karolinska Institutet, Sweden.

### Cell culture

Fresh mononuclear bone marrow cells were isolated with Lymphoprep® according to manufacturer's instructions, and separation of CD34+ progenitor cells was done with MACS® Microbeads technology. Cells were cultured with and without 1μM Aza (Celgene, New Jersey, US) in Erythroblast medium (containing Isocove's medium, BIT (15%), Rh SCF (25 ng/ml), Rh IL-3 (10 ng/ml), Rh IL-6 (10 ng/ml) and L-Glut 200 mm (1%)) in a humidified incubator with an atmosphere of 5% CO_2_ at 37°C, as previously described [4]. Aza was added at the start of the culture experiment. The cells were harvested at 0 h after separation, and after 24h and 48 h with and without exposure to Aza. Cells continuing to 48 h of culture were washed at 24 hours and then cultured in medium for another 24 h without addition of Aza. The cells were counted in a Neuberger chamber using Trypan blue (dilution 1:20). Cells were collected and centrifuged at 1500 rpm for 10 min. The supernatant was discarded and the pellet was then used for further applications. Cell growth was assessed by counting the number of cells present in the culture, calculating the relative difference of live cells at harvest compared to the amount of cells at time 0 h. Moreover, in a second independent experiment, vital frozen MNCs from five higher-risk MDS patients (of which two were the same patients as included in the culture experiment of primary fresh CD34+ cells) were thawed, ficoll separated and cultured for 24h with or without Aza (1 μM), where after CD34+ and CD34– cells were separated by magnetic beads as described above

### DNA/RNA extraction

Genomic DNA and RNA from CD34+ cells was extracted using the Allprep® kit, following manufacturer's instructions (Allprep DNA/RNA/Protein kit, Qiagen, Valencia, US). The concentration was measured with the Qubit® Assay based method (Thermo Fischer Scientific, Waltham, US).

### Mutational analyses

Eleven patients were analyzed for 42 genes recurrently mutated in myeloid disorders using Haloplex® target enrichment technology (Agilent Technologies, CA, US) followed by high throughput sequencing as previously described (Supplementary Table 1) [45].

### Methylation profiling

Bisulfite modified DNA from bone marrow CD34+ cells, separated and extracted according to the above, was processed using Illumina-supplied reagents and conditions and run on Illumina 450k bead chip arrays at the BEA core facility, Karolinska Institutet. Pre-processing and normalization used a pipeline reported previously [46] DNA methylation levels β (1 = fully methylated, 0 = fully demethylated) and M were defined as described [46, 47]. Briefly, the R packages lumi, methylumi, minfi and limma were used for quality control, normalization, and differential methylation analysis [47–50]. We filtered-out the following: 65 probes overlapping known SNPs in their sequence (allele frequency >1% in European population), probes on chromosomes X and Y and probes with a detection p value >0.01 exceeding 5% of the samples. Color-bias adjustment and quantile normalization were performed on pooled signal intensities, as implemented in lumi [47]. We then performed probe type bias adjustment using BMIQ normalization on β-values [51]. Differentially methylated probes (DMPs) were defined using limma on M-values, including the treatment (Aza vs. Control) and the patient as covariates [49]. We selected the following contrasts: Aza 24 h vs. control 24 h (effect of Aza treatment at 24 h) and control 24 h (effect of tissue culture at 24 h). For each contrast, coefficients (difference in M-values) p-values and false discovery rate (FDR) were estimated using an empirical Bayes method included in the limma package. DMP were selected if FDR<0.05. Gene-specific methylation for each gene was calculated as mean of all probes annotated to specific genes according to the Illumina annotation system. Similarly, gene specific promoter methylation was calculated including only promoter-associated probes.

Standard Illumina annotation was used to annotate methylation data, in addition to data on the epigenome of primary CD34+ cells, with 15 distinct chromatin states as defined by five histone marks (H3K4me3, H3K4me1, H3K27me3, H3K36me3, H3K9me3) in the Epigenome road map project (https://sites.google.com/site/anshulkundaje/projects/epigenomeroadmap) [52]. For each annotation category, the relative proportions of probes located within each feature type were calculated for DMPs, non-DMPs and the entire array.

### RNA sequencing and bioinformatics

The single cell–tagged reverse transcription (STRT) RNA-seq method with modifications was used to measure transcription initiation at the 5′end of polyA^+^ transcripts starting from 10 ng of total RNA as template [53, 54]. According to the 10 ng bulk RNA input, the cDNA was amplified by using 14 cycles of PCR and 10 additional cycles to introduce the complete sets of adapters for Illumina sequencing (Illumina). The library was size selected (200–400 bp) by using sequential Agencourt® AMPure® XP magnetic beads (Beckman Coulter, A63881, CA, US) selection protocol and 0.7× and 0.22× ratios, respectively. Preprocess alignment and differential expression tests were performed on UCSC hg19 reference genome by STRTprep which was an updated version (v3dev branch; https://github.com/shka/STRTprep/tree/v3dev) for quantitations by, not only protein-coding genes, but also transcript far 5′-ends (TFEs) [54, 55]. Overlap between TFEs and repetitive elements, which were provided by UCSC genome browser, was also examined [56]. Gene ontology (GO) analysis was performed using the GOrilla software [57].

### Chromatin immunoprecipitation followed by sequencing (ChIP seq)

CD34+ cells were treated as described above and 250 000 cells per condition were used for ChIP, using a 10% of each as Input. Cells were collected by centrifugation (5 min, 1600 rpm, 4°C) and resuspended in PBS (Invitrogen, city) up to 20 million cells/ml. Cells were fixed adding formaldehyde (37%, Sigma Aldrich, 252549, NJ, US) to the cell suspension to a final concentration of 1% with an incubation of 8 min at room temperature. Crosslinking was quenched by adding 2.5 M glycine to a final concentration of 125 mM, and cells were washed twice with ice cold PBS. ChIP was performed using the iDeal ChIP-Seq kit set for Histones (Diagenode, AB-001-0024, Liege, Belgium) following the manufacturer's instructions. For chromatin shearing, 300 μl of cell suspension in shearing buffer were transferred to 1.5 ml TPX microtubes (Diagenode, C30010010-300, Liege, Belgium), city) and sonicated using the Bioruptor sonicator (Diagenode, B01020001). The shearing protocol for CD34+ cells was optimized to 25 cycles of sonication (30s on, 30s off) with vortex and centrifugation step every 10 cycles to produce fragments 200–500 bp. For the magnetic immunoprecipitation, 1 μl of ChIP-seq grade control antibody was added per tube with 100 μl of sheared chromatin. The antibodies used were: H3K9Me3 (Abcam, ab8988) andH3K18Ac (Abcam, ab1191).

After decrosslinking, the fragmented DNA was purified with the MiniElute PCR Purification Kit (Qiagen, 28004) and the DNA concentration was measured using Qubit Fluorometric Quantitation with High Sensitivity (Thermo Fischer Scientific, Waltham, US, Q32851). The library was prepared using the ThruPLEX DNA-seq Kit with single indexes (Rubicon Genomics, Ann Arbor, MI, R400428) following the instructions of the manufacturer. The quality of samples was subsequently evaluated using the 2100 Bioanalyzer system (Agilent, Santa Clara, CA) and the library clean-up and size selection was performed using AMPure® XP magnetic beads (Beckman Coulter). DNA concentration was measured using Qubit (Thermo Fischer Scientific, Waltham, US, Q32851). Samples were barcoded and up to four samples per lane were pooled.

After pooling of libraries, libraries were hybridized to the flow cell and sequenced on the Illumina HiSeq2000 with single end sequencing. As a primary quality control of the sequencing the intensities and base calling parameters were checked (using default parameter settings) with the HiSeq Control software, followed by de-multiplexing of the libraries.

### Bioinformatic analysis of ChIP sequencing data

The raw sequencing reads in FASTQ format were quality checked with the FASTQC tool (Babraham Bioinformatics, Cambridge, UK, version 0.11.4). Adapter sequences and duplicates were removed. We aimed for at least10 million filtered reads. The filtered data was then aligned with Bowtie2 (version 2.2.6) to the Human Reference Genome Issue HG-19 (https://www.ncbi.nlm.nih.gov/grc/human/issues/HG-19) with the requirement of unique alignment. Bedgraphs were generated using BED tools (BEDtool v 2.16.2) and data was visualized as custom tracks within the UCSC genome browser (https://genome.ucsc.edu). Peak calling was performed with HOMER (v4.8, 1-13-2016, http://homer.salk.edu.homer/) to determine enrichment over the input samples across the whole genome. Peak size was optimized to 1000 bp, with the minimum distance to the next peak set to 2500 bp. The closest transcription start site (TSS) was then annotated to the enriched regions using HOMER, and differential analysis between treated and control samples were performed with edgeR (Bioconductor version 3.14.0, https://bioconductor.org/packages/release/bioc/html/edgeR). For analysis of correlation of ChIP seq data to RNA seq and DNA methylation data, raw filtered data were mapped to the human reference genome as above (HG-19, Bowtie2). Thereafter we calculated tags within 100 000 bp from TSS and annotated this peak to Ref Seq (https://www.ncbi.nlm.nih.gov/refseq) to find genes in the corresponding positions. Thereafter, differences between Aza treated and control sample were assessed in EdgeR as fold change, which was used for correlation comparison to RNA seq and DNA methylation data.

### Motif finding analysis for gene expression data

Promoter transcription factor (TF) binding motif analysis was performed for genes with significantly increased gene expression at 24 h in Aza samples compared to untreated controls, with the Homer Motif analysis algorithm (version 4.8, 1-13-2016). The algorithm analyzed gene promoters for motifs enriched in our target gene promoters relative to known human gene promoters. Parameters used for the analysis were the input file containing the gene ID of target genes, background promote set (HG-19), start and end position relative to the transcription start site (−start −400 -end 100 to TSS and motif length set to 8, 10 and 12 nucleotides.

## SUPPLEMENTARY MATERIALS FIGURES AND TABLES




