# Supplementary material for: Comprehensive mapping of the effects of azacitidine on DNA methylation, repressive/permissive histone marks and gene expression in primary cells from patients with MDS and MDS-related disease
Source: Oncotarget. 2017 Feb 28;8(17):28812–25. doi: 10.18632/oncotarget.15807 (PMC5438694; doi:10.18632/oncotarget.15807)
Supplement: Supplementary file 1 [file oncotarget-08-28812-s001.pdf]

Comprehensive mapping of the effects of Azacitidine on DNA methylation, repressive / permissive histone marks and gene expression in primary cells from patients with MDS and MDS-related disease

SUPPLEMENTARY FIGURES AND TABLES

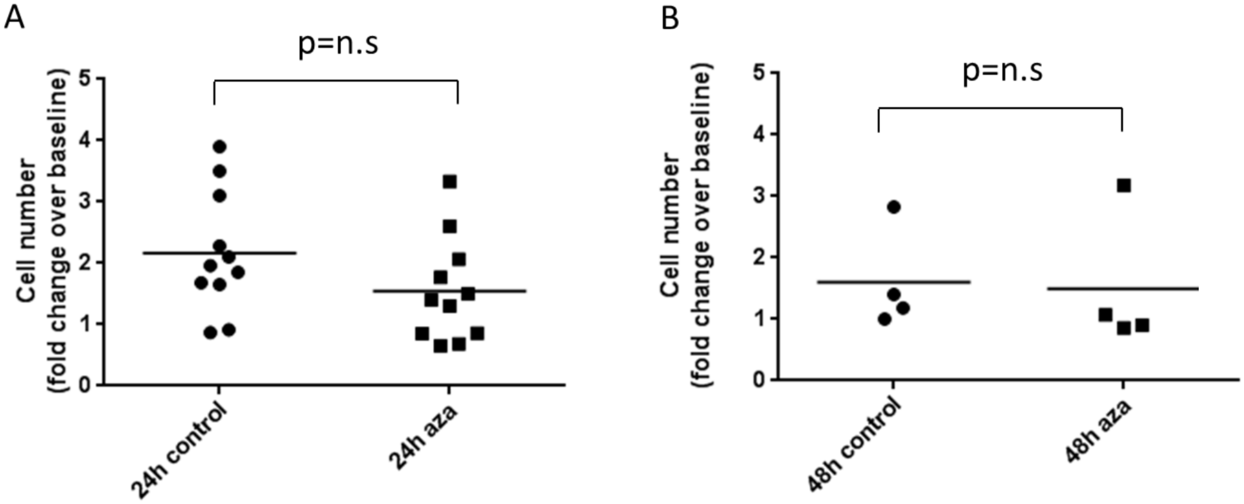

Supplementary Figure 1: Cell growth for CD34+ progenitor cells: (A) 24h of culture (B) 48h of culture.

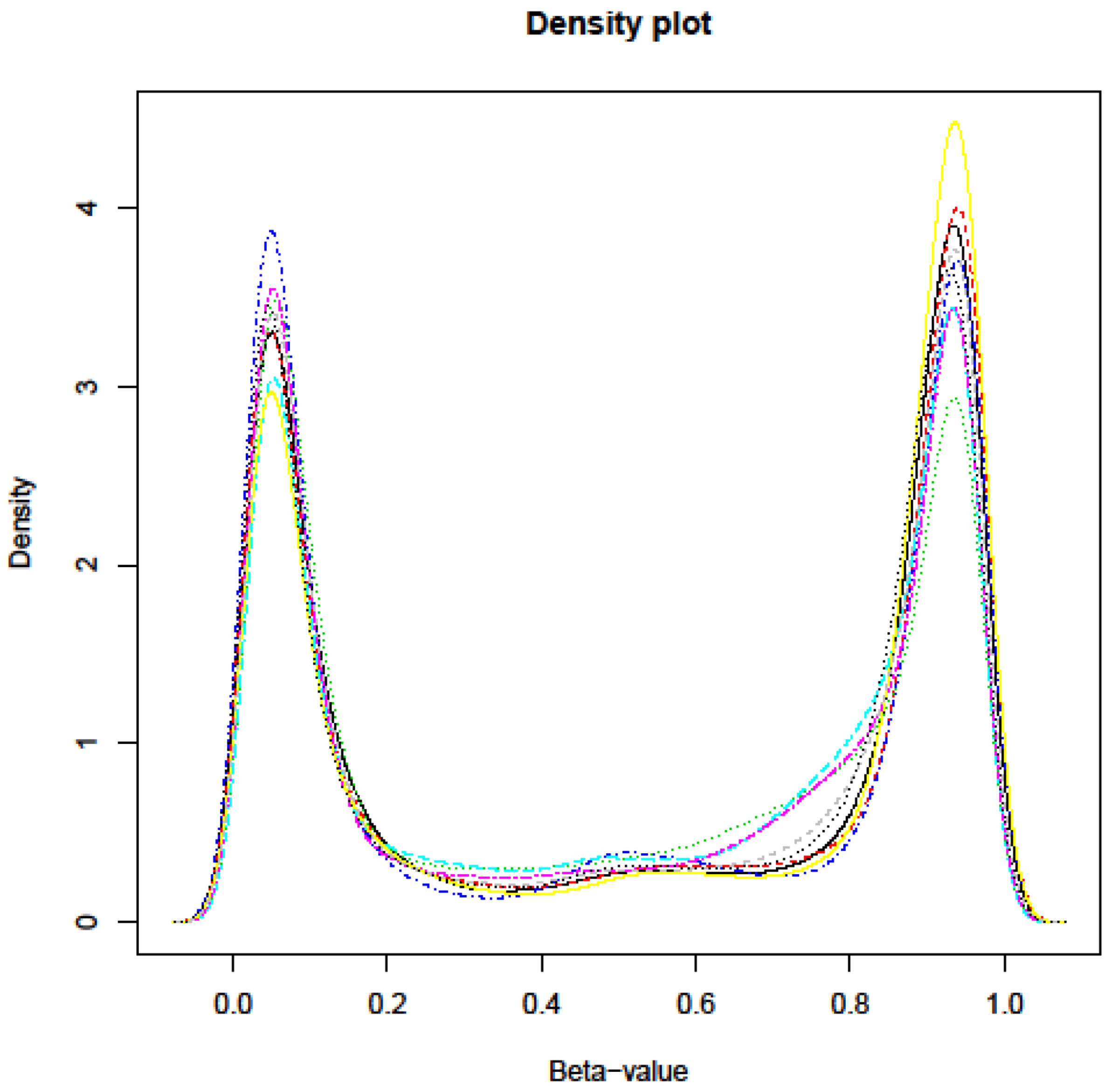

Supplementary Figure 2: Density plot of DNA methylation beta-values across the samples.

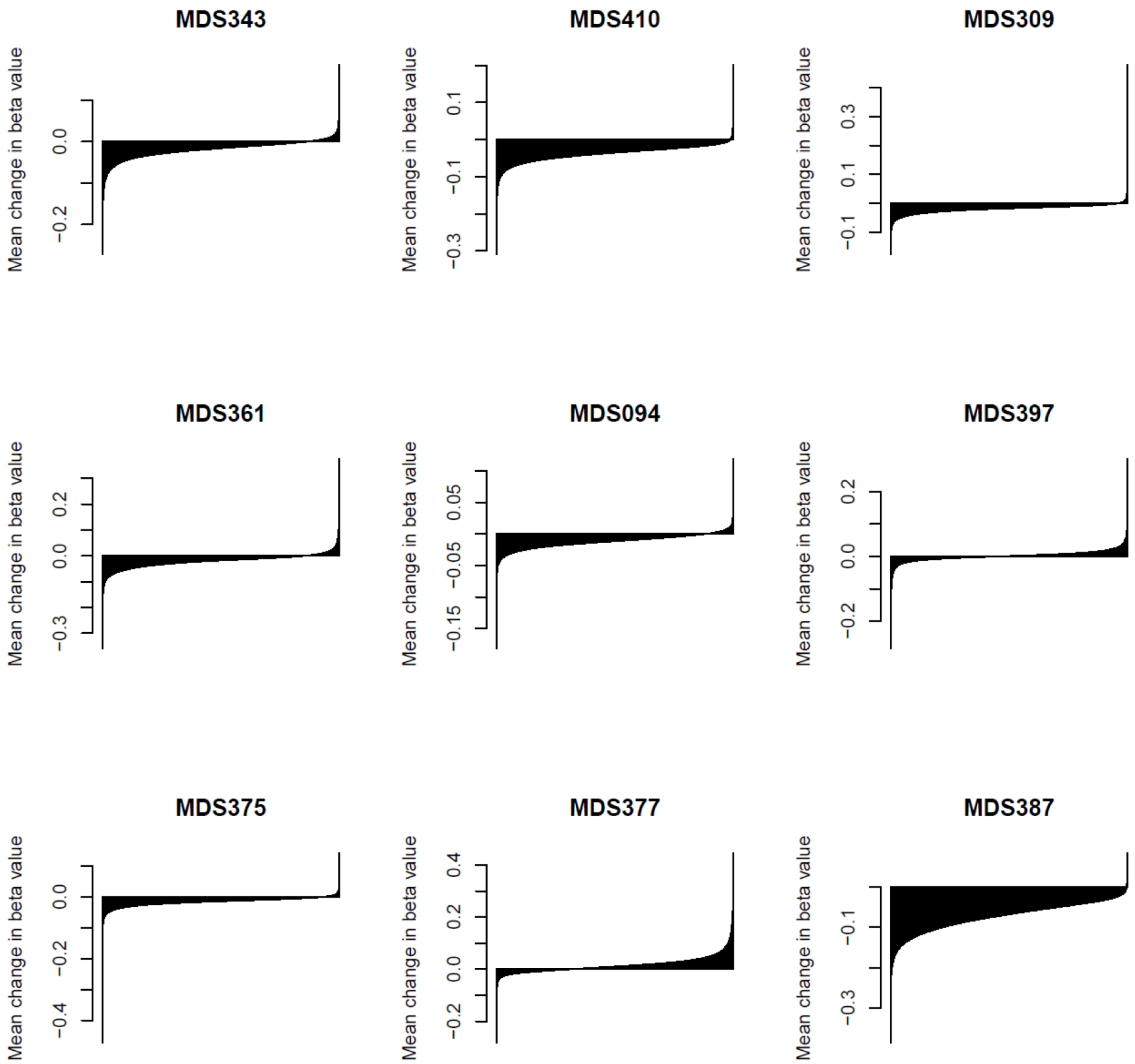

Supplementary Figure 3: Mean change in methylation for all probes annotated for specific genes; all genes sorted from most negatively to positively changed and printed as a barplot for each patient specifically.

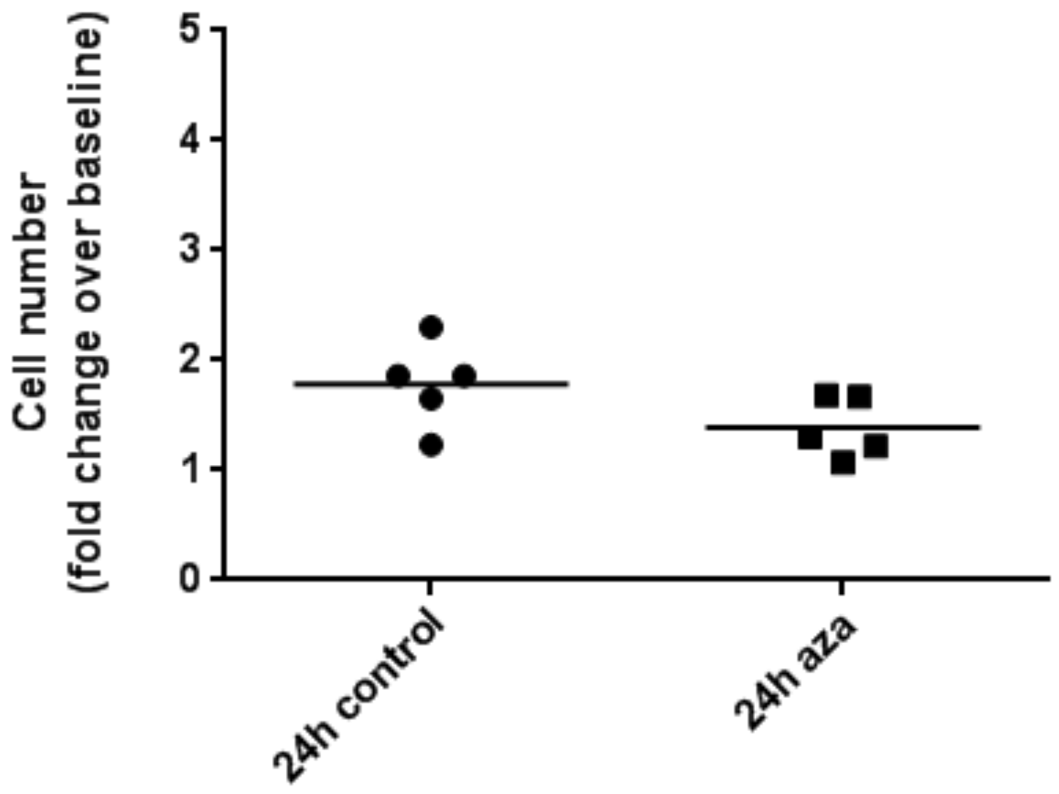

Supplementary Figure 4: Cell growth for MNC cells.

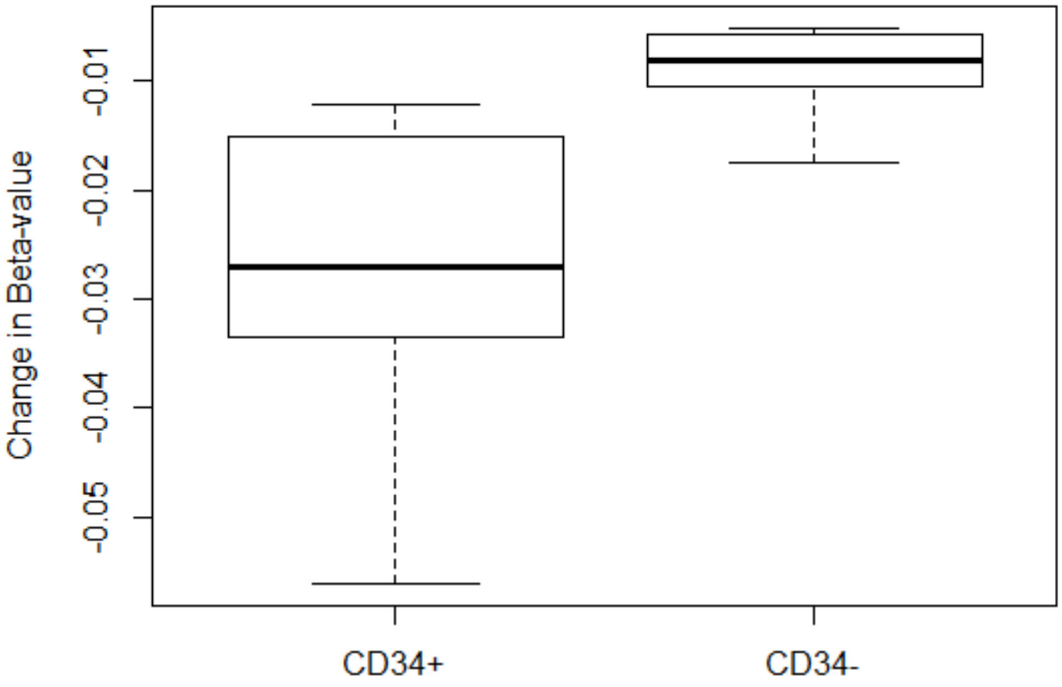

Supplementary Figure 5: Change in DNA methylation in CD34+ and CD34- cells.

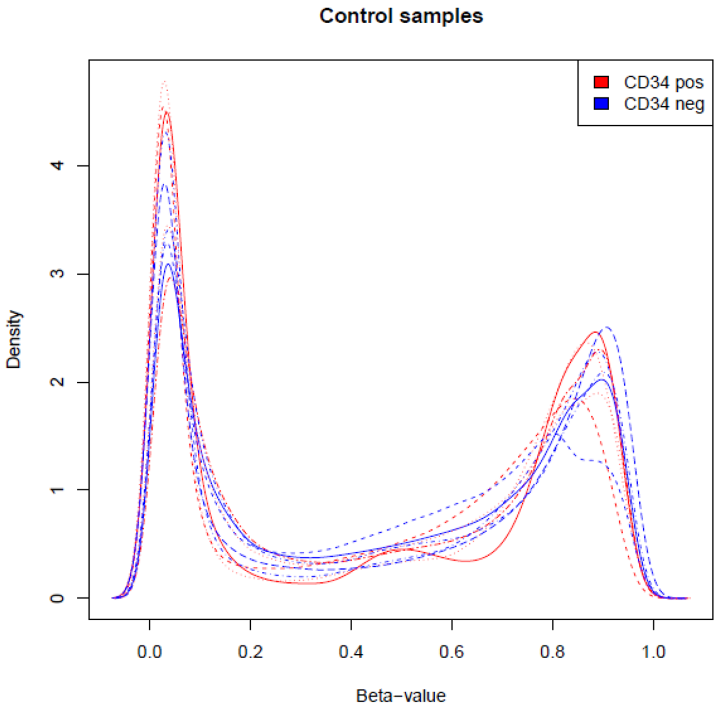

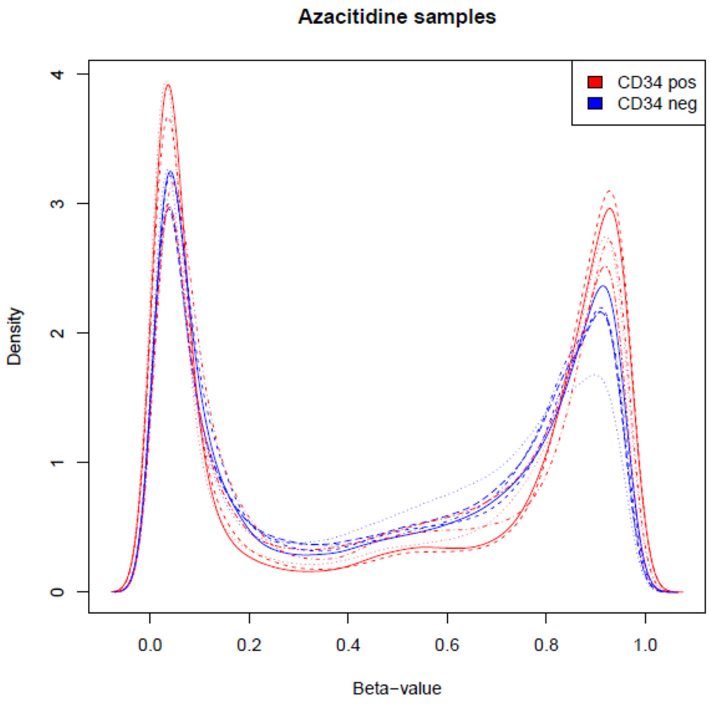

Supplementary Figure 6: Density plot for DNA methylation in CD34 pos / neg cells in treated / untreated samples.

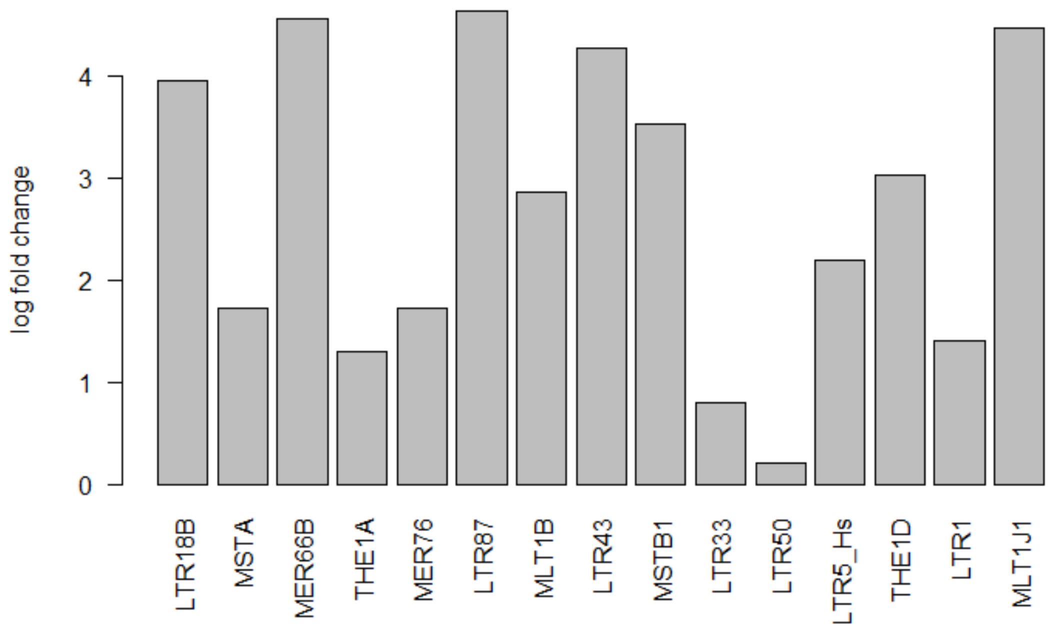

Supplementary Figure 7: Activated ERVs in Aza-treated samples.

Supplementary Table 1: Genes included in the mutational panel

ASXL1	GATA1	MPL	SH2B3	WT1
BCOR	GATA2	NPM1	SMC1A	ZRSR2
CBL	GATA3	NRAS	SMC3	
CEBPA	IDH1	PDS5B	SRSF2	
CSF3R	IDH2	PRPF40B	STAG1	
DNMT3A	JAK2	RAD21	STAG2	
EPOR	KDM6A	RUNX1	TET2	
ETV6	KIT	SF1	TP53	
EZH2	KRAS	SF3A1	U2AF1	
FLT3	MLL	SF3B1	U2AF2	

Supplementary Table 2: Enriched GO pathways for genes upregulated in Aza samples compared to control samples.

See Supplementary File 1

Supplementary Table 3: Correlation between gene expression and the epigenetic modalities. Each cells in the table indicate p-value and rho-value

Patient	DNA methylation / gene expression	Gene expression / H3K9me3	Gene expression / H3K18ac	DNA methylation / H3K9me3	DNA methylation / H3K18ac	H3K9me3 / H3K18ac
UPN1				3e-192 / -0.22		
UPN2	9e-01 / 0.001	2e-03 / -0.03	2e-02 / 0.026	8e-53 / 0.115	3e-01 / 0.007	7e-01 / 0.002
UPN4				2e-69 / 0.132		
UPN5	2e-02 / -0.028	6e-01 / -0.006		3e-117 / -0.172		
UPN6	8e-01 / -0.004	3e-01 / 0.012	7e-01 / 0.004	5e-04 / -0.026	4e-03 / -0.023	7e-01 / 0.003
UPN11	8e-02 / -0.02	1e-04 / -0.043		3e-24 / 0.076		
Global	2e-01 / -0.014	2e-07 / -0.053	1 / 0	2e-69 / 0.136	9e-01 / 0.001	2e-01 / 0.007

Supplementary Table 4: Activated ERVs

LTRs	LTR family	TFE	Gene	Location	Region	Str	p-value	fold change
LTR18B	ERVL	TFE44558	chr6:29932811-29932997;+	Unannotated	chr6:29932811-29932997	+	0.003	51.697
MSTA	ERVL-MaLR	TFE48100	UPK3B	Coding 3'-UTR	chr7:76157030-76157096	+	0.008	5.625
MER66B	ERV1	TFE13477	chr12:121540132-121540172;-	Unannotated	chr12:121540132-121540172	-	0.009	95.706
THE1A	ERVL-MaLR	TFE50126	chr8:55074627-55074725;+	Unannotated	chr8:55074627-55074725	+	0.014	3.647
MER76	ERVL	TFE24091	RFNG	Coding 3'-UTR	chr17:80006042-80006567	-	0.016	5.620
LTR87	ERVL	TFE25511	MLLT1	Coding 3'-UTR	chr19:6210989-6211202	-	0.023	102.628
MLT1B	ERVL-MaLR	TFE13020	chr12:105086800-105087018;-	Unannotated	chr12:105086800-105087018	-	0.027	17.579
LTR43	ERV1	TFE28514	ERVV-1	Coding 3'-UTR	chr19:53519551-53519666	+	0.029	71.857
MSTB1	ERVL-MaLR	TFE42036	chr5:49949867-49949998;-	Unannotated	chr5:49949867-49949998	-	0.032	34.155
LTR33	ERVL	TFE50912	PVT1	Intron	chr8:129061580-129061855	+	0.034	2.213
LTR50	ERVL	TFE51083	LOC100507316	Noncoding 1st-exon	chr8:144363706-144363876	-	0.041	1.231
LTR5_Hs	ERVK	TFE12764	chr12:85863364-85863427;-	Unannotated	chr12:85863364-85863427	-	0.041	8.933
THE1D	ERVL-MaLR	TFE35736	LINC01399	Noncoding upstream	chr22:35627012-35627053	-	0.048	20.708
LTR1	ERV1	TFE45769	chr6:78078748-78078886;-	Unannotated	chr6:78078748-78078886	-	0.049	4.083
MLT1J1	ERVL-MaLR	TFE36306	RRP7A	Coding 3'-UTR	chr22:42904616-42904739	-	0.050	87.414
