# Supplementary material for: Comprehensive mapping of the effects of azacitidine on DNA methylation, repressive/permissive histone marks and gene expression in primary cells from patients with MDS and MDS-related disease
Source: Oncotarget. 2017 Feb 28;8(17):28812–25. doi: 10.18632/oncotarget.15807 (PMC5438694; doi:10.18632/oncotarget.15807)
Supplement: Supplementary file 2 [file oncotarget-08-28812-s002.docx]

**Table S2: Enriched GO pathways for genes upregulated in Aza samples compared to control samples**

| **GO process** |  |  |  |  |
| --- | --- | --- | --- | --- |
| GO term | Description | [P-value](http://cbl-gorilla.cs.technion.ac.il/GOrilla/ccmnkpd8/GOResultsPROCESS.html#p_value_info) | [FDR q-value](http://cbl-gorilla.cs.technion.ac.il/GOrilla/ccmnkpd8/GOResultsPROCESS.html#fdr_info) | [Enrichment (N, B, n, b)](http://cbl-gorilla.cs.technion.ac.il/GOrilla/ccmnkpd8/GOResultsPROCESS.html#enrich_info) |
| [GO:0006413](http://www.godatabase.org/cgi-bin/amigo/go.cgi?query=GO:0006413&view=details) | translational initiation | 8.37E-11 | 9.40E-07 | 2.80 (7152,125,981,48) |
| [GO:0034660](http://www.godatabase.org/cgi-bin/amigo/go.cgi?query=GO:0034660&view=details) | ncRNA metabolic process | 2.02E-10 | 1.14E-06 | 1.91 (7152,383,985,101) |
| [GO:0072599](http://www.godatabase.org/cgi-bin/amigo/go.cgi?query=GO:0072599&view=details) | establishment of protein localization to endoplasmic reticulum | 4.41E-10 | 1.65E-06 | 3.08 (7152,90,981,38) |
| [GO:0045047](http://www.godatabase.org/cgi-bin/amigo/go.cgi?query=GO:0045047&view=details) | protein targeting to ER | 6.21E-10 | 1.74E-06 | 3.10 (7152,87,981,37) |
| [GO:0006396](http://www.godatabase.org/cgi-bin/amigo/go.cgi?query=GO:0006396&view=details) | RNA processing | 6.85E-10 | 1.54E-06 | 1.70 (7152,581,985,136) |
| [GO:0070972](http://www.godatabase.org/cgi-bin/amigo/go.cgi?query=GO:0070972&view=details) | protein localization to endoplasmic reticulum | 7.08E-10 | 1.33E-06 | 2.99 (7152,95,981,39) |
| [GO:0006613](http://www.godatabase.org/cgi-bin/amigo/go.cgi?query=GO:0006613&view=details) | cotranslational protein targeting to membrane | 2.03E-09 | 3.26E-06 | 3.00 (7152,90,981,37) |
| [GO:0016071](http://www.godatabase.org/cgi-bin/amigo/go.cgi?query=GO:0016071&view=details) | mRNA metabolic process | 2.44E-09 | 3.43E-06 | 1.83 (7152,411,981,103) |
| [GO:0006614](http://www.godatabase.org/cgi-bin/amigo/go.cgi?query=GO:0006614&view=details) | SRP-dependent cotranslational protein targeting to membrane | 2.68E-09 | 3.34E-06 | 3.07 (7152,83,981,35) |
| [GO:0016072](http://www.godatabase.org/cgi-bin/amigo/go.cgi?query=GO:0016072&view=details) | rRNA metabolic process | 3.40E-09 | 3.82E-06 | 2.21 (7152,204,985,62) |
| [GO:0000184](http://www.godatabase.org/cgi-bin/amigo/go.cgi?query=GO:0000184&view=details) | nuclear-transcribed mRNA catabolic process, nonsense-mediated decay | 5.94E-09 | 6.07E-06 | 2.82 (7152,101,981,39) |
| [GO:0034470](http://www.godatabase.org/cgi-bin/amigo/go.cgi?query=GO:0034470&view=details) | ncRNA processing | 6.24E-09 | 5.84E-06 | 1.98 (7152,290,985,79) |
| [GO:0019083](http://www.godatabase.org/cgi-bin/amigo/go.cgi?query=GO:0019083&view=details) | viral transcription | 6.56E-09 | 5.67E-06 | 2.90 (7152,93,981,37) |
| [GO:0006412](http://www.godatabase.org/cgi-bin/amigo/go.cgi?query=GO:0006412&view=details) | translation | 7.84E-09 | 6.29E-06 | 2.28 (7152,173,996,55) |
| [GO:0043043](http://www.godatabase.org/cgi-bin/amigo/go.cgi?query=GO:0043043&view=details) | peptide biosynthetic process | 1.09E-08 | 8.20E-06 | 2.25 (7152,179,996,56) |
| [GO:0006364](http://www.godatabase.org/cgi-bin/amigo/go.cgi?query=GO:0006364&view=details) | rRNA processing | 1.51E-08 | 1.06E-05 | 2.17 (7152,201,985,60) |
| [GO:0043604](http://www.godatabase.org/cgi-bin/amigo/go.cgi?query=GO:0043604&view=details) | amide biosynthetic process | 5.05E-08 | 3.34E-05 | 2.12 (7152,200,996,59) |
| [GO:0006612](http://www.godatabase.org/cgi-bin/amigo/go.cgi?query=GO:0006612&view=details) | protein targeting to membrane | 4.26E-07 | 2.66E-04 | 2.47 (7152,115,981,39) |
| [GO:0006518](http://www.godatabase.org/cgi-bin/amigo/go.cgi?query=GO:0006518&view=details) | peptide metabolic process | 5.52E-07 | 3.26E-04 | 1.96 (7152,227,996,62) |
| [GO:0000956](http://www.godatabase.org/cgi-bin/amigo/go.cgi?query=GO:0000956&view=details) | nuclear-transcribed mRNA catabolic process | 2.71E-06 | 1.53E-03 | 2.20 (7152,146,981,44) |
| [GO:0033365](http://www.godatabase.org/cgi-bin/amigo/go.cgi?query=GO:0033365&view=details) | protein localization to organelle | 3.30E-06 | 1.76E-03 | 1.76 (7152,311,981,75) |
| [GO:0043603](http://www.godatabase.org/cgi-bin/amigo/go.cgi?query=GO:0043603&view=details) | cellular amide metabolic process | 3.43E-06 | 1.75E-03 | 1.80 (7152,275,996,69) |
| [GO:0072594](http://www.godatabase.org/cgi-bin/amigo/go.cgi?query=GO:0072594&view=details) | establishment of protein localization to organelle | 8.37E-06 | 4.09E-03 | 1.85 (7152,232,981,59) |
| [GO:0006402](http://www.godatabase.org/cgi-bin/amigo/go.cgi?query=GO:0006402&view=details) | mRNA catabolic process | 1.15E-05 | 5.37E-03 | 2.10 (7152,153,981,44) |
| [GO:0006605](http://www.godatabase.org/cgi-bin/amigo/go.cgi?query=GO:0006605&view=details) | protein targeting | 1.67E-05 | 7.52E-03 | 1.91 (7152,195,981,51) |
| [GO:0090150](http://www.godatabase.org/cgi-bin/amigo/go.cgi?query=GO:0090150&view=details) | establishment of protein localization to membrane | 3.72E-05 | 1.61E-02 | 1.94 (7152,169,981,45) |
| [GO:0034622](http://www.godatabase.org/cgi-bin/amigo/go.cgi?query=GO:0034622&view=details) | cellular macromolecular complex assembly | 3.87E-05 | 1.61E-02 | 1.57 (7152,421,983,91) |
| [GO:1901566](http://www.godatabase.org/cgi-bin/amigo/go.cgi?query=GO:1901566&view=details) | organonitrogen compound biosynthetic process | 5.31E-05 | 2.13E-02 | 1.56 (7152,419,996,91) |
| [GO:0000462](http://www.godatabase.org/cgi-bin/amigo/go.cgi?query=GO:0000462&view=details) | maturation of SSU-rRNA from tricistronic rRNA transcript (SSU-rRNA, 5.8S rRNA, LSU-rRNA) | 5.50E-05 | 2.13E-02 | 3.96 (7152,22,985,12) |
| [GO:0016070](http://www.godatabase.org/cgi-bin/amigo/go.cgi?query=GO:0016070&view=details) | RNA metabolic process | 7.96E-05 | 2.98E-02 | 1.24 (7152,1741,951,288) |
| [GO:1902582](http://www.godatabase.org/cgi-bin/amigo/go.cgi?query=GO:1902582&view=details) | single-organism intracellular transport | 8.36E-05 | 3.03E-02 | 1.66 (7152,294,996,68) |
| [GO:0046907](http://www.godatabase.org/cgi-bin/amigo/go.cgi?query=GO:0046907&view=details) | intracellular transport | 1.17E-04 | 4.12E-02 | 1.41 (7152,686,981,133) |
|  |  |  |  |  |
| **GO Function** |  |  |  |  |
| GO term | Description | [P-value](http://cbl-gorilla.cs.technion.ac.il/GOrilla/ccmnkpd8/GOResultsFUNCTION.html#p_value_info) | [FDR q-value](http://cbl-gorilla.cs.technion.ac.il/GOrilla/ccmnkpd8/GOResultsFUNCTION.html#fdr_info) | [Enrichment (N, B, n, b)](http://cbl-gorilla.cs.technion.ac.il/GOrilla/ccmnkpd8/GOResultsFUNCTION.html#enrich_info) |
| [GO:0003723](http://www.godatabase.org/cgi-bin/amigo/go.cgi?query=GO:0003723&view=details) | RNA binding | 2.55E-12 | 8.14E-09 | 1.55 (7152,1065,983,227) |
| [GO:0044822](http://www.godatabase.org/cgi-bin/amigo/go.cgi?query=GO:0044822&view=details) | poly(A) RNA binding | 2.03E-10 | 3.24E-07 | 1.58 (7152,825,997,182) |
| [GO:0003735](http://www.godatabase.org/cgi-bin/amigo/go.cgi?query=GO:0003735&view=details) | structural constituent of ribosome | 7.10E-08 | 7.56E-05 | 2.23 (7152,164,996,51) |
| [GO:0003676](http://www.godatabase.org/cgi-bin/amigo/go.cgi?query=GO:0003676&view=details) | nucleic acid binding | 1.39E-05 | 1.11E-02 | 1.23 (7152,2029,983,344) |
|  |  |  |  |  |
|  |  |  |  |  |
| **GO Component** |  |  |  |  |
| GO term | Description | [P-value](http://cbl-gorilla.cs.technion.ac.il/GOrilla/ccmnkpd8/GOResultsCOMPONENT.html#p_value_info) | [FDR q-value](http://cbl-gorilla.cs.technion.ac.il/GOrilla/ccmnkpd8/GOResultsCOMPONENT.html#fdr_info) | [Enrichment (N, B, n, b)](http://cbl-gorilla.cs.technion.ac.il/GOrilla/ccmnkpd8/GOResultsCOMPONENT.html#enrich_info) |
| [GO:1990904](http://www.godatabase.org/cgi-bin/amigo/go.cgi?query=GO:1990904&view=details) | ribonucleoprotein complex | 3.23E-11 | 4.78E-08 | 1.79 (7152,525,997,131) |
| [GO:0030529](http://www.godatabase.org/cgi-bin/amigo/go.cgi?query=GO:0030529&view=details) | intracellular ribonucleoprotein complex | 3.23E-11 | 2.39E-08 | 1.79 (7152,525,997,131) |
| [GO:0044391](http://www.godatabase.org/cgi-bin/amigo/go.cgi?query=GO:0044391&view=details) | ribosomal subunit | 5.85E-07 | 2.88E-04 | 2.31 (7152,139,981,44) |
| [GO:0044445](http://www.godatabase.org/cgi-bin/amigo/go.cgi?query=GO:0044445&view=details) | cytosolic part | 1.28E-06 | 4.74E-04 | 2.18 (7152,155,994,47) |
| [GO:0032991](http://www.godatabase.org/cgi-bin/amigo/go.cgi?query=GO:0032991&view=details) | macromolecular complex | 3.10E-06 | 9.17E-04 | 1.22 (7152,2466,981,411) |
| [GO:0022625](http://www.godatabase.org/cgi-bin/amigo/go.cgi?query=GO:0022625&view=details) | cytosolic large ribosomal subunit | 6.77E-06 | 1.67E-03 | 3.03 (7152,53,980,22) |
| [GO:0044446](http://www.godatabase.org/cgi-bin/amigo/go.cgi?query=GO:0044446&view=details) | intracellular organelle part | 6.86E-06 | 1.45E-03 | 1.13 (7152,4065,983,631) |
| [GO:0044422](http://www.godatabase.org/cgi-bin/amigo/go.cgi?query=GO:0044422&view=details) | organelle part | 1.39E-05 | 2.56E-03 | 1.12 (7152,4110,983,635) |
| [GO:0071013](http://www.godatabase.org/cgi-bin/amigo/go.cgi?query=GO:0071013&view=details) | catalytic step 2 spliceosome | 4.12E-05 | 6.77E-03 | 2.52 (7152,74,997,26) |
| [GO:0005681](http://www.godatabase.org/cgi-bin/amigo/go.cgi?query=GO:0005681&view=details) | spliceosomal complex | 7.82E-05 | 1.16E-02 | 2.08 (7152,138,948,38) |
| [GO:0044424](http://www.godatabase.org/cgi-bin/amigo/go.cgi?query=GO:0044424&view=details) | intracellular part | 1.66E-04 | 2.23E-02 | 1.05 (7152,6098,999,895) |
